# LRRK2 inhibition does not impart protection from α-synuclein pathology and neuron death in non-transgenic mice

**DOI:** 10.1186/s40478-019-0679-5

**Published:** 2019-02-26

**Authors:** Michael X. Henderson, Medha Sengupta, Ian McGeary, Bin Zhang, Modupe F. Olufemi, Hannah Brown, John Q. Trojanowski, Virginia M. Y. Lee

**Affiliations:** Department of Pathology and Laboratory Medicine, Institute on Aging and Center for Neurodegenerative Disease Research, University of Pennsylvania School of Medicine, 3600 Spruce St, 3rd Floor Maloney, Philadelphia, PA 19104-4283 USA

**Keywords:** Leucine-rich repeat kinase 2, pS129, Aggregation, Inhibitor, G2019S, MLi-2

## Abstract

Mutations in leucine-rich repeat kinase 2 (*LRRK2*) are one of the most common causes of familial Parkinson’s disease (PD). The most common mutations in the *LRRK2* gene induce elevated kinase activity of the LRRK2 protein. Recent studies have also suggested that LRRK2 kinase activity may be elevated in idiopathic PD patients, even in the absence of *LRRK2* mutations. LRRK2 is therefore a prime candidate for small molecule kinase inhibitor development. However, it is currently unknown how LRRK2 influences the underlying pathogenesis of PD and how LRRK2 might influence extant pathogenesis. To understand whether LRRK2 inhibition would show some benefit in the absence of LRRK2 mutations, we treated a preclinical mouse model of PD with the potent LRRK2 inhibitor MLi-2. The inhibitor was well-tolerated by mice and dramatically reduced LRRK2 kinase activity. However, LRRK2 inhibition did not reverse motor phenotypes, pathological α-synuclein accumulation or neuron loss. The current study suggests that LRRK2 is not necessary for α-synuclein pathogenesis in this mouse model of PD and that further studies are needed to assess the likely clinical benefit of LRRK2 inhibition in idiopathic PD.

## Introduction

Parkinson’s disease (PD) is the second most common neurodegenerative disease, after Alzheimer’s disease (AD), and the most common neurodegenerative movement disorder. PD patients are characterized initially by movement difficulties, but as high as 80% of PD patients will go on to develop dementia as well [19]. Neurons in the olfactory bulb and brainstem are affected first, causing the characteristic motor and olfactory deficits, and over time disease spreads to higher cortical areas concurrent with cognitive decline [4–6]. However, the underlying etiology of this degeneration is still not well understood.

While PD is largely sporadic, insight has been gained through investigation of some of the rare familial mutations that lead to PD. Several of the early mutations identified, as well as whole gene duplications and triplications were in the gene encoding the synaptic protein α-synuclein [2, 7, 18, 20, 21, 27–29, 31, 38]. This is the same protein that forms the characteristic pathology Lewy body aggregates seen in PD [15, 32, 33], suggesting that α-synuclein is a key protein in disease pathogenesis. One of the most commonly mutated genes in PD is leucine-rich repeat kinase 2 (*LRRK2*) [17]. This large protein has scaffolding, GTPase and kinase domains. Interestingly, the most common mutation in *LRRK2* in the kinase domain (p.G2019S), and mutations outside of the kinase domain all seem to elevate kinase activity of the protein [16, 30, 34, 37]. This finding would suggest that elevated LRRK2 kinase activity is playing a role in PD pathogenesis, and has led pharmaceutical companies to develop many highly selective and potent inhibitors of LRRK2 activity for the treatment of PD.

More recently, there has been evidence from urinary exosomes [12] and the brains of idiopathic PD patients [8], that even in the absence of *LRRK2* mutations, LRRK2 kinase activity may be elevated. If LRRK2 kinase activity is indeed elevated in idiopathic PD patients, it would suggest that LRRK2 is driving some aspect of PD pathogenesis and that LRRK2 inhibitors may be efficacious even in patients that do not carry mutations. Indeed, LRRK2 inhibitor administration in a rat neurotoxin model of degeneration prevented accumulation of pathological α-synuclein [8]. A separate study showed that antisense oligonucleotides (ASOs), which reduce the levels of LRRK2, were also able to reduce the amount of pathological α-synuclein in the vulnerable substantia nigra of mice inoculated with pathological α-synuclein [39]. If inhibiting LRRK2 kinase activity or reducing total LRRK2 levels are efficient at reducing α-synuclein pathology, this may be a viable therapeutic avenue for all patients with PD and even other synucleinopathies.

We have recently developed a mouse model that exhibits α-synuclein pathology throughout the brain and vulnerable neuron death without the overexpression of α-synuclein [23]. Treatment of these mice with the potent LRRK2 inhibitor MLi-2 has allowed us to directly assess the tolerability of LRRK2 inhibition, the extent of LRRK2 kinase inhibition, motor behavior, α-synuclein pathology and neuron death. We report here that MLi-2 is well-tolerated in mice and shows effective inhibition of LRRK2 kinase activity both peripherally and in the central nervous system. However, mice treated with the inhibitor showed no improvement in motor performance, similar development of α-synuclein pathology and similar levels of dopaminergic neuron death compared to control animals. We find that LRRK2 is not essential to α-synuclein pathogenesis in PD and suggest that further studies are necessary to determine whether LRRK2 inhibition will be a viable therapeutic for idiopathic PD.

## Materials and methods

### Animals

All housing, breeding, and procedures were performed according to the NIH Guide for the Care and Use of Experimental Animals and approved by the University of Pennsylvania Institutional Animal Care and Use Committee. All mice used in this study were C57BL/6J (JAX 000664, RRID: IMSR_JAX:000664).

### Behavior

Mouse all-limb grip strength was measured using the animal grip strength test (IITC 2200). For this test a rod is attached to a digital force transducer. Mice are moved to a quiet behavioral testing suite and allowed to acclimate for 1 h. Each mouse is held by the base of the tail and allowed to grasp the rod. Once the mouse clasps the rod, the mouse is slowly moved backwards, in line with the force transducer until the mouse releases the rod. The maximum grip force is recorded. The mouse is allowed to rest for several seconds, and then placed on the rod again. The maximum grip strength of 5 tests was recorded. No fatigue was observed during the test period, so the average of all 5 measures is reported.

An accelerating rotarod (MED-Associates) was used to assess motor coordination. Mice received two training sessions and two tests sessions. During the training sessions, mice were placed on a still rod. The rod then began to accelerate from 4 rotations per minute (rpm) to 40 rpm over 5 min. Mice were allowed to rest at least 1 h between training and testing sessions. During the testing sessions, mice were treated as before, and the latency to fall was recorded. The trial was also concluded if a mouse gripped the rod and rotated with it instead of walking. Mice were allowed a maximum of 10 min on the rod.

### MLi-2 administration

Mice were assigned to control (*n* = 7) or MLi-2 (*n* = 8) groups to equally match sex. Administration of MLi-2 or control diet began 3 days prior to α-synuclein PFF injection. Control diet (Research Diets D01060501) was the same as the MLi-2 diet (Research Diets D17031301) except for the compound and a dye to allow visual discrimination between the two diets. MLi-2 was incorporated at 240 mg/kg diet to achieve approximately 30 mg/kg/day dosing based on approximately 3–4 g diet consumption/day in mice that were approximately 25 g and stayed the same weight for the course of the study. This dose was selected based on previous work with this molecule [11]. Mice were weighed once a week and diet was weighed and replenished every 2–3 days to allow assessment of estimated dosage.

### α-synuclein PFF PD mouse model

Purification of recombinant α-synuclein and generation of α-synuclein PFFs was conducted as described elsewhere [24, 35, 36]. All surgery experiments were performed in accordance with protocols approved by the Institutional Animal Care and Use Committee (IACUC) of the University of Pennsylvania. Mouse α-synuclein PFFs, which were generated at a concentration of 5 mg/mL were vortexed and diluted with Dulbecco’s phosphate-buffered saline (DPBS) to 2 mg/mL. They were then sonicated on high for 10 cycles of 30 s on, 30 s off (Diagenode Biorupter UCD-300 bath sonicator).

Mice were injected when 3–4 months old. Mice were injected unilaterally by insertion of a single needle into the right forebrain (coordinates: + 0.2 mm relative to Bregma, + 2.0 mm from midline) targeting the dorsal striatum (2.6 mm beneath the dura) with 5 μg α-synuclein PFFs (2.5 μL). Injections were performed using a 10 μL syringe (Hamilton, NV) at a rate of 0.4 μL/minute. After 3 months, mice were perfused transcardially with PBS, brains were removed and underwent overnight fixation in 70% ethanol in 150 mM NaCl, pH 7.4. Kidney and lung were removed and underwent overnight fixation in 10% neutral buffered formalin. Spinal cord and liver were snap frozen and stored at − 80 °C for biochemistry.

### Immunohistochemistry

After perfusion and fixation, brains were embedded in paraffin blocks, cut into 6 μm sections and mounted on glass slides. Slides were then stained using standard immunohistochemistry as described below. Slides were de-paraffinized with 2 sequential 5-min washes in xylenes, followed by 1-min washes in a descending series of ethanols: 100, 100, 95, 80, 70%. Slides were then incubated in deionized water for 1 min prior to antigen retrieval as noted. After antigen retrieval, slides were incubated in 5% hydrogen peroxide in methanol to quench endogenous peroxidase activity. Slides were washed for 10 min in running tap water, 5 min in 0.1 M tris, then blocked in 0.1 M tris/2% fetal bovine serum (FBS). Slides were incubated in primary antibodies overnight. The following primary antibodies were used. For pathologically-phosphorylated α-synuclein, pS129 α-synuclein (EP1536Y; Abcam ab51253, RRID:AB_869973) was used at 1:20,000 with microwave antigen retrieval with citric acid based antigen unmasking solution (Vector H-3300, RRID:AB_2336226). To stain midbrain dopaminergic neurons, Tyrosine hydroxylase (TH-16; Sigma-Aldrich T2928, RRID:AB_477569) was used at 1:5000 with formic acid antigen retrieval. To stain pneumocytes, anti-prosurfactant protein C (proSP-C, Millipore Sigma AB3786, RRID:AB_91588) was used at 1:4000 with microwave antigen retrieval as described above.

Primary antibody was rinsed off with 0.1 M tris for 5 min, then incubated with goat anti-rabbit (Vector BA1000, RRID:AB_2313606) or horse anti-mouse (Vector BA2000, RRID:AB_2313581) biotinylated IgG in 0.1 M tris/2% FBS 1:1000 for 1 h. Biotinylated antibody was rinsed off with 0.1 M tris for 5 min, then incubated with avidin-biotin solution (Vector PK-6100, RRID:AB_2336819) for 1 h. Slides were then rinsed for 5 min with 0.1 M tris, then developed with ImmPACT DAB peroxidase substrate (Vector SK-4105, RRID:AB_2336520) and counterstained briefly with Harris Hematoxylin (Fisher 67–650-01). Slides were washed in running tap water for 5 min, dehydrated in ascending ethanol for 1 min each: 70, 80, 95, 100, 100%, then washed twice in xylenes for 5 min and coversliped in Cytoseal Mounting Media (Fisher 23–244-256).

Hematoxylin and eosin staining was performed on kidney and lung sections using a standard protocol. Briefly, sections were immersed in Harris Hematoxylin (Fisher 67–650-01) for 1 min, rinsed 2 times in distilled water, differentiated in 0.1% acid alcohol solution for 4 s and rinsed in tap water for 15 min. Sections were then immersed in eosin for 1 min, briefly rinsed in tap water, then dehydrated and mounted with Cytoseal Mounting Media (Fisher 23–244-256).

Slides were scanned into digital format on a Lamina scanner (Perkin Elmer) at 20× magnification. Digitized slides were then used for quantitative pathology.

### Quantitative histology

All section selection, annotation and quantification was done blinded to genotype. All quantitation was performed in HALO quantitative pathology software (Indica Labs). Every 10th slide through the midbrain was stained with tyrosine hydroxylase (TH). TH-stained sections were used to annotate the substantia nigra, and cell counting was performed manually in a blinded manner for all sections. The sum of all sections was multiplied by 10 to estimate the total count that would be obtained by counting every section. The substantia nigra annotations drawn onto the TH-stained sections were then transferred to sequential sections that had been stained for pS129 α-synuclein. A single analysis algorithm was then applied equally to all stained sections to quantify the percentage of area occupied by pS129 α-synuclein staining. Specifically, the analysis included all DAB signal that was above a 0.157 optical density threshold, which was empirically determined to not include any background signal. This signal was then normalized to the total tissue area. A minimal tissue optical density of 0.02 was used to exclude any areas where tissue was split. Four other regions were selected for α-synuclein pathology quantification based on their susceptibility to pathology changes in mice expressing G2019S LRRK2 (data not shown). For these regions, only two sections flanking − 2.92 mm relative to Bregma were selected for each brain. The noted regions were annotated on these sections and quantified using the same algorithm as noted above.

### Biochemistry

Spinal cords and livers were thawed on ice and suspended in 5× volumes/weight of tissue RIPA buffer (50 mM Tris, pH 7.6, 150 mM NaCl, 1% NP-40, 0.5% sodium deoxycholate, 0.1% SDS, 1 mM EDTA, 10 mM NaF) with proteinase and phosphatase inhibitors and 1 mM PMSF. Total protein concentration in each sample was determined by a bicinchoninic acid colorimetric assay (Fisher 23,223 and 23,224), using bovine serum albumin as a standard (Thermo Fisher 23,210). 180 μg of total liver or spinal cord protein was resolved on 5–20% gradient acrylamide gels. Western Blot analysis was performed with primary antibodies targeting LRRK2 (ab133474, Abcam, RRID: AB_2713963, 1:500), pS935 LRRK2 (ab133450, Abcam, RRID:AB_2732035, 1:400) or GAPDH (2-RGM2, Advanced Immunological, RRID:AB_2721282, 1:5000). Primary antibodies were detected using IRDye 800 (Li-cor 925–32,210) or IRDye 680 (Li-cor 925–68,071) secondary antibodies, scanned on Li-cor Odyssey Imaging System and analyzed using Image Studio software. LRRK2 and pS935 LRRK2 values were normalized to GAPDH as an internal loading control, then further normalized to the mean of all control samples.

### Statistical analysis

All statistical analyses were done in GraphPad Prism 7. The analysis used for each data set is described in the figure legends.

## Results

### MLi-2 is well tolerated and allows for relatively steady dosing in mice

We have recently developed a mouse model of PD that does not rely on the overexpression of α-synuclein or neurotoxins, but rather injection of a small amount of pathogenic α-synuclein seeds in the dorsal striatum of wildtype mice [23]. These mice develop pathological pS129 α-synuclein inclusions that spread throughout the mouse brain to anatomically-interconnected regions, including the vulnerable substantia nigra. Pathology increases over time for 3 months, after which time the dopaminergic neurons of the nigra degenerate and pathology is reduced proportionally [23]. The pathological spread and neuron death in these mice recapitulate important features of PD, and allows the assay of LRRK2 inhibition in a wildtype context. For our studies, we chose to use the potent and specific LRRK2 inhibitor MLi-2 [11]. This inhibitor has a reported IC_50_ of 0.76 nM and greater than 295-fold selectivity for over 300 kinases [11]. In addition to these properties, MLi-2 is also orally bioavailable, brain penetrant and is stable in mice over prolonged periods of time.

**Fig. 1:**
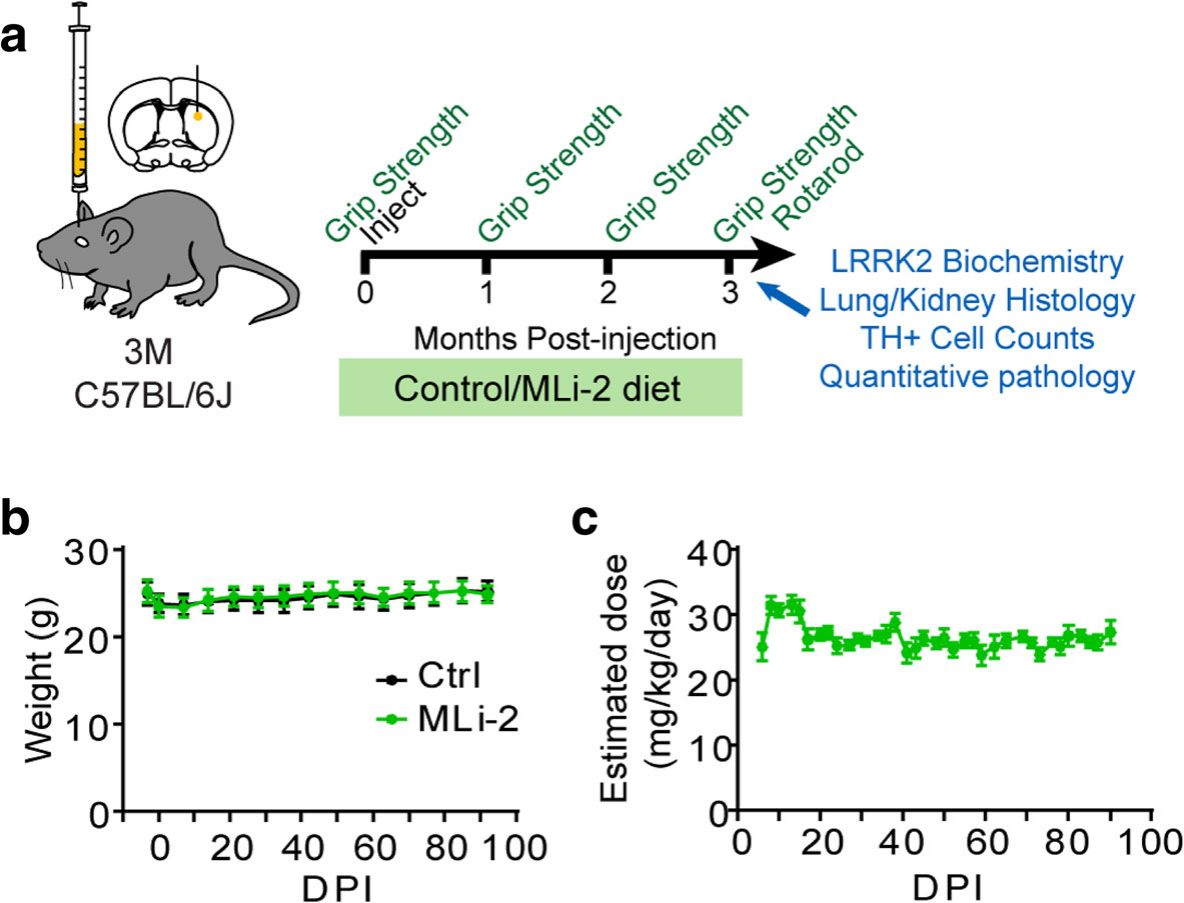
Wildtype mice injected with α-synuclein PFFs tolerate LRRK2 inhibition with MLi-2. **a** Experimental design schematic. C57BL/6J mice aged 3 months were evaluated at baseline for grip strength, then treated with MLi-2 in diet or control diet 3 days prior to injection with α-synuclein PFFs. After injection, mice were aged a further 3 months, during which time they received constant exposure to control or MLi-2 treatment and were assessed for grip strength every month. At 3 months, mice were given terminal assessment on both grip strength and accelerating rotarod, then sacrificed and analyzed as described. **b** Mouse body weight was assessed once weekly during the study and was steady over the course of the study in both groups, following a slight drop after surgery. **c** Based on mouse body weights and estimated diet consumption from diet weights, mice received steady dosing during the study, with an average estimated dosage of 26.5 mg/kg/day. Plots are means +/− s.e.m.

In the current study, mice of 3 months of age were assayed for baseline grip strength so their motor function could be assayed over the course of the study (Fig. 1a). A 3-month trial duration was chosen based on the fact that mice in this paradigm will have extensive pathology and degeneration of dopaminergic substantia nigra neurons at this time point. Based on the initial description of MLi-2, we decided to incorporate MLi-2 into diet at a concentration of 240 mg/kg with the goal of achieving 30 mg/kg/day dosage which was shown to almost completely inhibit wildtype LRRK2 in mice [11]. Mice were switched from their regular chow to either control diet or MLi-2 diet 3 days prior to injection with α-synuclein PFFs and kept on this diet for the entirety of the 3-month treatment period (Fig. 1a).

Mice tolerated the diet well and showed no difference in weight compared to control diet animals (Fig. 1b). Diet was weighed every 2–3 days. Combined with mouse weights, diet weight was used to estimate dosage of MLi-2 during the study (Fig. 1c). After initial overconsumption following surgery, mice maintained steady diet consumption for the duration of the study, with an estimated mean dose of 26.5 mg/kg/day.

### MLi-2 inhibits LRRK2 kinase activity in the periphery and central nervous system

**Fig. 2:**
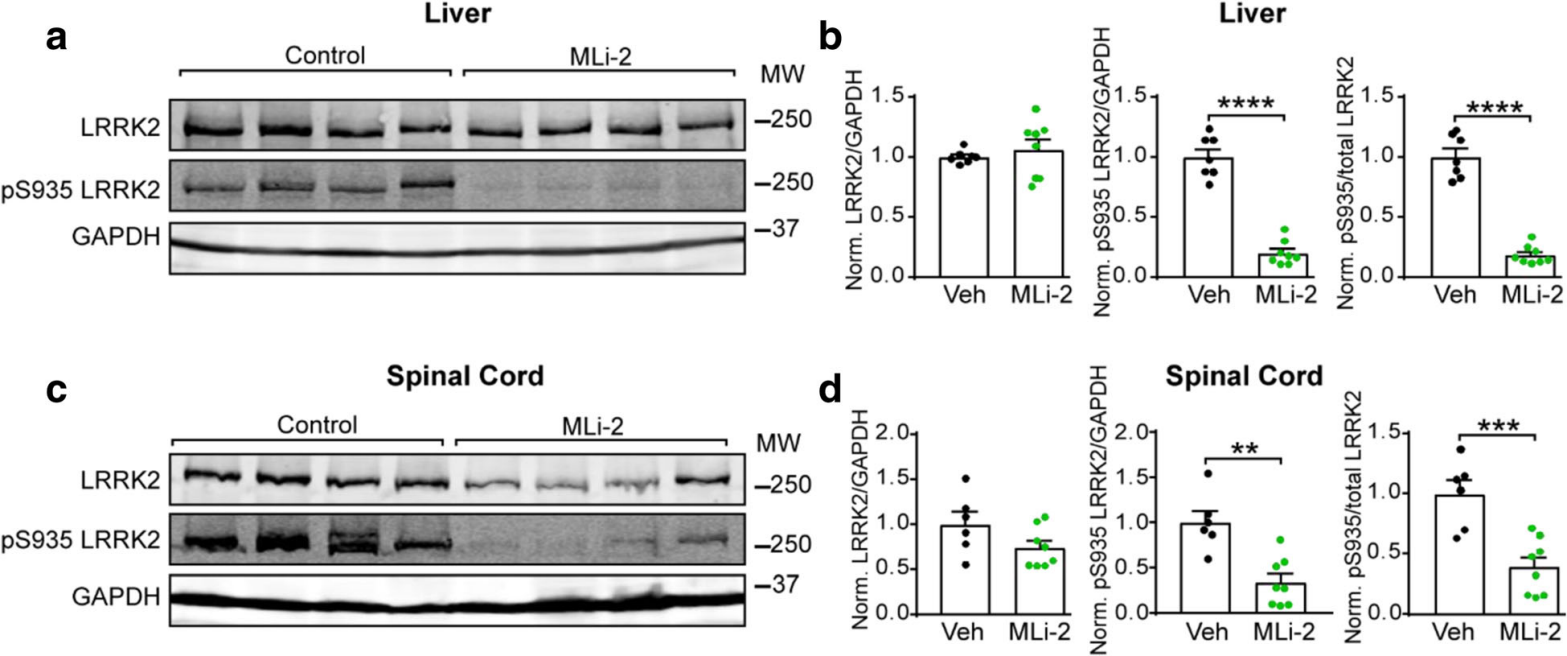
Chronic MLi-2 administration results in dramatic LRRK2 inhibition peripherally and in the central nervous system of mice. **a** Western blot images from liver lysate of animals treated with control or MLi-2 diet and assayed for total LRRK2, pS935 LRRK2 and GAPDH levels. **b** Quantification of total LRRK2 and pS935 LRRK2 levels normalized to GAPDH as an internal control and the mean of all control diet-treated animals. LRRK2, *p* = 0.4891, t test with Welch’s correction; pS935 LRRK2; *p* < 0.0001, unpaired t test; pS935/total LRRK2, *p* < 0.0001, unpaired t test. **c** Western blot images from spinal cord lysate of animals treated with control or MLi-2 diet and assayed for total LRRK2, pS935 LRRK2 and GAPDH levels. **d** Quantification of total LRRK2 and pS935 LRRK2 levels normalized to GAPDH as an internal control and the mean of all control diet-treated animals. LRRK2, *p* = 0.0989, unpaired t test; pS935 LRRK2; *p* = 0.0011, unpaired t test; pS935/total LRRK2, *p* = 0.0008, unpaired t test. All plots are means and error bars represent s.e.m. with individual values plotted

Since mouse brains were fixed for histological analysis, spinal cords were utilized for biochemistry to allow assessment of central nervous system LRRK2 activity. The liver was used to assess peripheral LRRK2 activity. Both total LRRK2 and pS935 LRRK2 were detected by immunoblotting to determine LRRK2 kinase activity. While pS935 is not directly phosphorylated by LRRK2, it is a widely-used and reliable readout for LRRK2 kinase activity [8, 9, 11, 30]. MLi-2 treatment did not affect liver LRRK2 levels but reduced pS935 LRRK2 levels over 75% (Fig. 2a, b). MLi-2 treatment reduced total LRRK2 in the spinal cord by a mean of 26% and pS935 LRRK2 by over 65% (Fig. 2c, d). Thus, chronic dosing of MLi-2 in mice is able to achieve robust inhibition of LRRK2 kinase activity in both the periphery and central nervous system.

### LRRK2 inhibition leads to mild pneumocyte enlargement

**Fig. 3:**
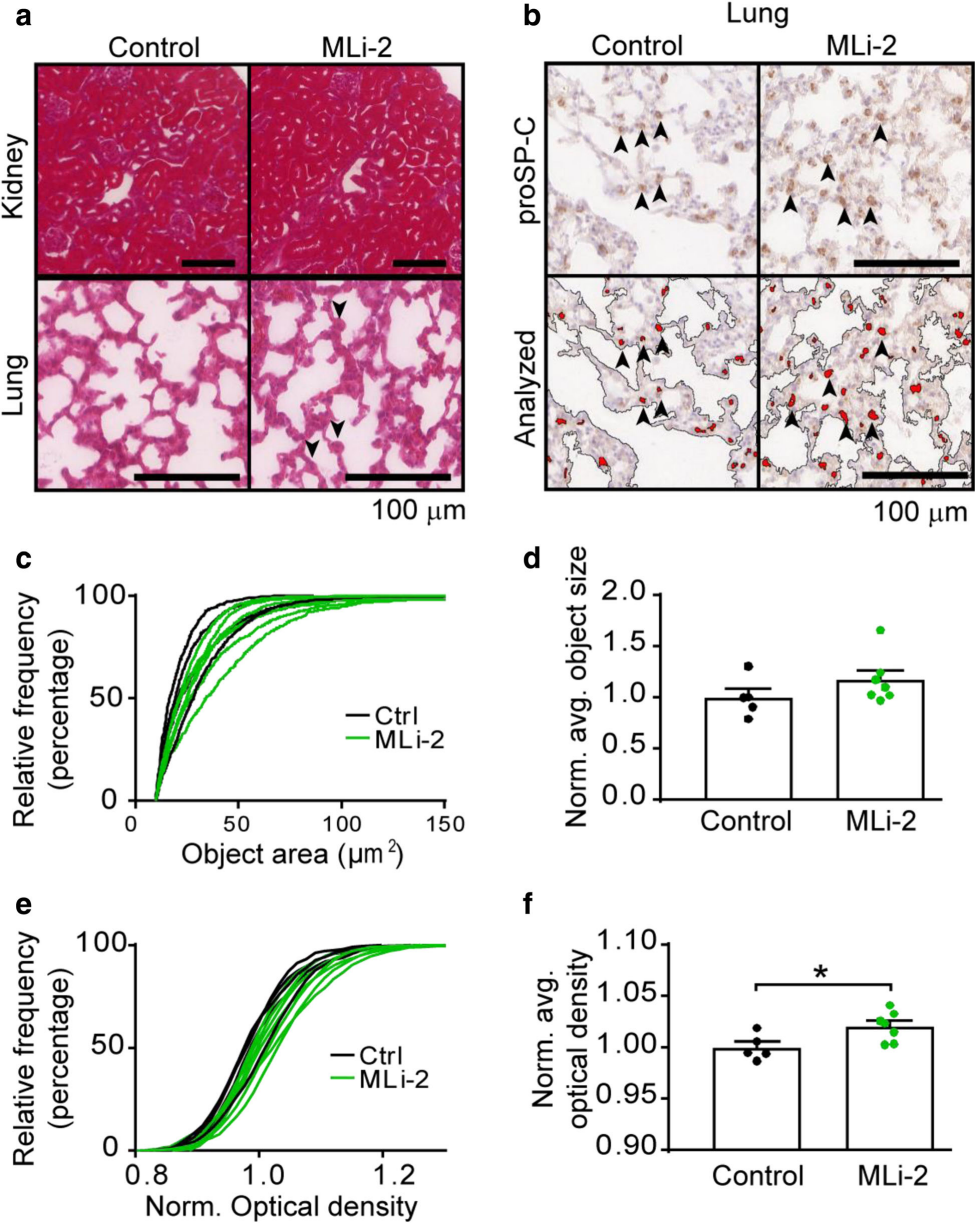
Chronic MLi-2 causes no apparent changes in kidney histology but causes enlargement of pneumocytes in the lung. **a** Hematoxylin and eosin-stained kidney and lung sections are shown from control and MLi-2 treated animals. There was no apparent change in kidney histology in these animals, but in the lung there appeared to be several enlarged alveolar epithelial cells (arrowheads). **b** To further characterize whether these cells were type II pneumocytes, lung sections were stained with an antibody recognizing prosurfactant protein C (proSP-C). A portion of these cells appear to be larger in MLi-2 treated lungs than in control treated lungs. A threshold analysis script was then applied to the tissue (analyzed). **c** The relative frequency of proSP-C positive objects is shifted rightward with MLi-2 treatment for several animals, though the overall average object size is not significantly increased (**d**, *p* = 0.2103, unpaired t test). **e** The relative frequency of optical density (OD) of proSP-C objects is also right shifted with MLi-2 treatment and the average OD of objects is increased (**f**, *p* = 0.0279, unpaired t test). Scale bars = 100 μm. The relative frequencies of individual mice are shown in (**c**) and (**e**). All plots are means and error bars represent s.e.m. with individual values plotted

Some LRRK2 inhibitors have previously been shown to cause histological abnormalities in mice [11, 13], rats [1] and non-human primates [13]. These abnormalities are not thought to be off-target effects due to the appearance of similar histological findings in LRRK2 knockout rats [3] and mice treated with LRRK2 ASOs [39]. Despite these histological findings, no measured physiological deficit has been found in these animal models or in participants in a clinical trial. Based on these reports, kidney and lung histology was performed. Hematoxylin and eosin staining did not reveal any abnormalities in the kidney, but there seemed to be enlargement of some alveolar epithelial cells within the lung (Fig. 3a). To further examine this enlargement lung sections were stained with an antibody which recognizes prosurfactant protein C (proSP-C), a marker of type II pneumocytes (Fig. 3b). This allowed observation and quantification of type II pneumocytes. The proSP-C positive objects in MLi-2 treated mice had a slightly larger size (Fig. 3c, d) and greater optical density (Fig. 3e, f), consistent with previous reports of MLi-2 [11] and other LRRK2 inhibitors [13].

### LRRK2 inhibition does not attenuate motor deficits following α-synuclein PFF injection

**Fig. 4:**
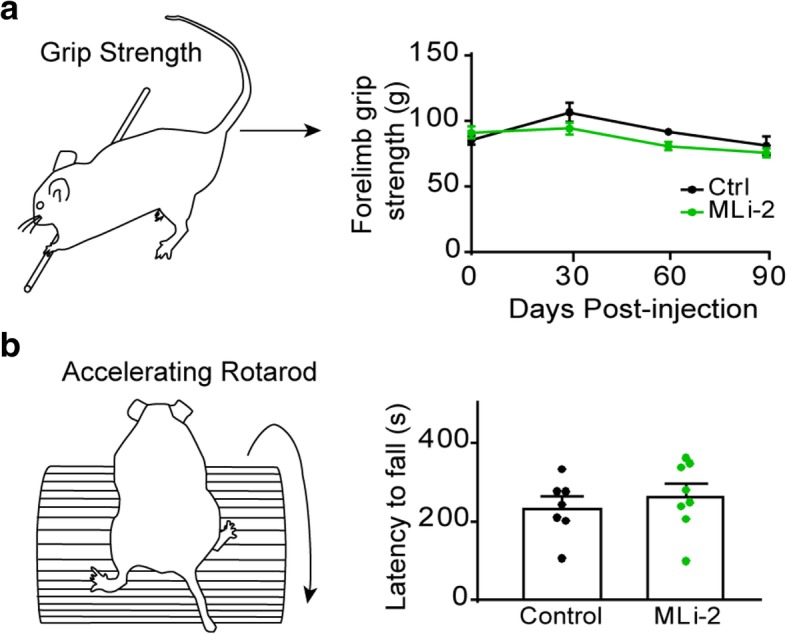
Motor behavior is not improved by chronic MLi-2 administration. **a** Mice were assayed for forelimb grip strength using a rod attached to a force transducer (see schematic) which recorded maximum force as the mouse is slowly moved backwards away from the rod. The average of five trials/mouse is reported. Mice slowly lose grip strength after α-synuclein PFF injection, but no effect of treatment with MLi-2 was observed (two-way ANOVA, source of variation: time, *p* = 0.0006; treatment, *p* = 0.0933. **b** 3 months after α-synuclein PFF injection, motor coordination was assayed using an accelerating rotarod in which the mouse is placed on a rod which slowly accelerates (see schematic). Control and MLi-2 treated animals showed no difference in latency to fall on this test (*p* = 0.4912, unpaired t test). Plots are means and error bars represent s.e.m. with individual values plotted

The pathology induced by α-synuclein PFF injection leads to neuron death in the nigrostriatal circuit and can lead to progressive motor weakness and incoordination [23]. Motor strength was assessed using a grip strength assay whereby mice are allowed to grip a bar with their forelimbs and are slowly pulled away from the apparatus while a force transducer records the maximum grip strength (Fig. 4a). Both control and MLi-2 treated animals show diminished motor strength over the course of the study, but there was no difference in the rate of decline between groups. Motor coordination as assayed by latency to fall on an accelerating rotarod was also not different between groups (Fig. 4b).

### LRRK2 inhibition does not protect neurons from α-synuclein pathology accumulation or neuron death

**Fig. 5:**
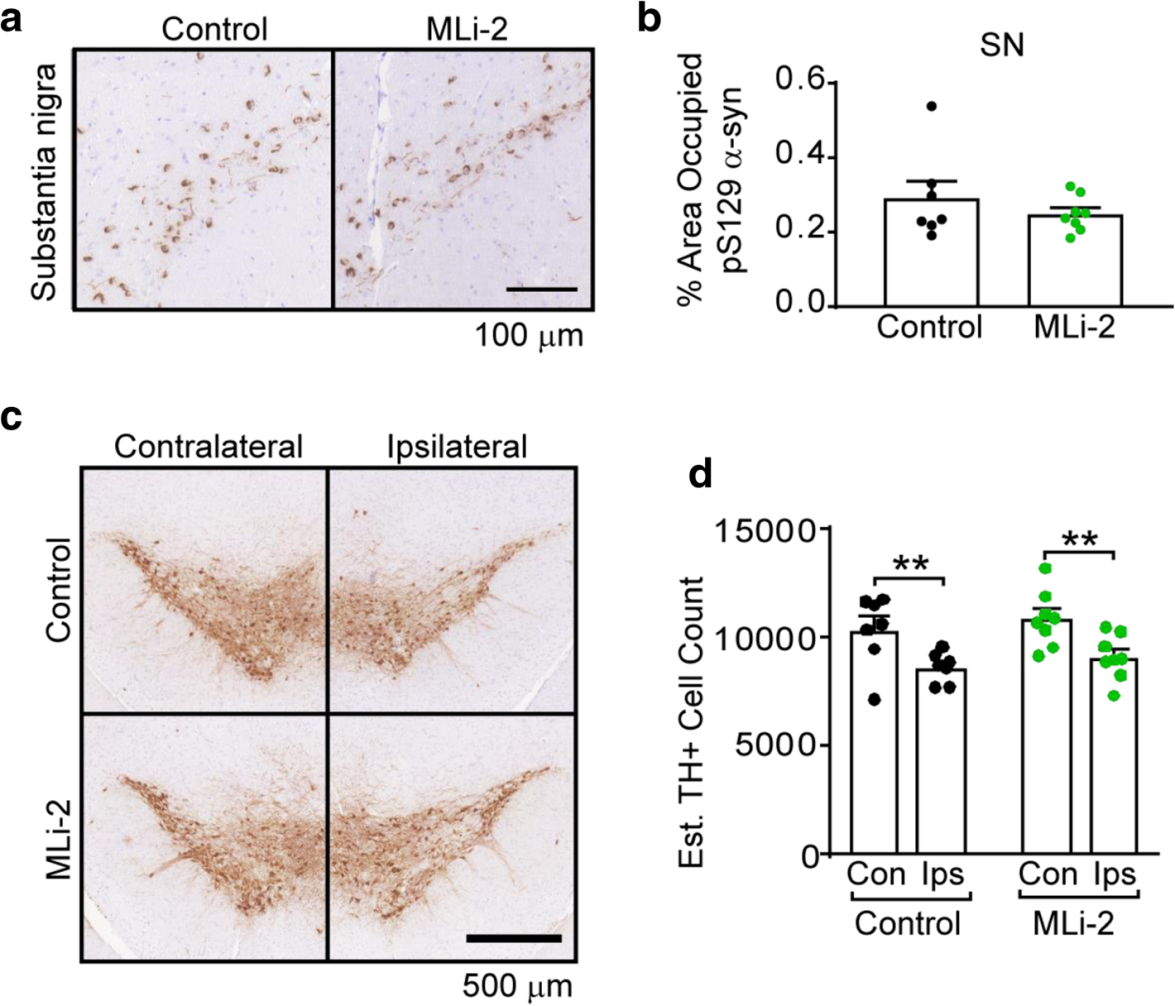
MLi-2 treatment does not impart protection from α-synuclein pathology or neuron death. **a** Both control and MLi-2 treated mice accumulate pS129 α-synuclein aggregates in the substantia nigra ipsilateral to the site of injection. Scale bars = 100 μm. **b** Quantification of pS129 α-synuclein pathology in every 10th section through the ipsilateral substantia nigra reveals that there is no difference between pathological burden in control and MLi-2 mice (*p* = 0.3995, unpaired t test with Welch’s correction). **c** The accumulation of α-synuclein pathology leads to substantial loss of substantia nigra neurons in both control and MLi-2 treated mice ipsilateral to the site of injection. This is shown here through use of TH staining. Scale bar = 500 μm. **d** Every 10th section through the midbrain was stained with TH and TH-positive cells were counted to estimate the total number of neurons in the substantia nigra. While there was significant loss of neurons ipsilateral to the site of injection (control *p* = 0.0048, MLi-2 *p* = 0.0026, two-way ANOVA followed by Sidak’s multiple comparisons test), there was no significant difference between control and MLi-2 treated mice (control versus MLi-2 ipsilateral to injection *p* = 0.6988, two-way ANOVA followed by Sidak’s multiple comparisons test). All plots are means and error bars represent s.e.m. with individual values plotted

Injection of α-synuclein PFFs into the dorsal striatum of mice leads to robust α-synuclein pathology in anatomically-connected regions, and a commensurate death of vulnerable dopaminergic neurons in the substantia nigra [23]. This phenotype allows us to directly assay the role of LRRK2 in the development and toxicity of α-synuclein pathology in wildtype mice. Every 10th section through the midbrain of mice was stained for pS129 α-synuclein and the percentage of the substantia nigra occupied by α-synuclein pathology was quantified (Fig. 5a, b). Robust pathology accumulated in both groups of mice. However, if LRRK2 is effecting pathogenesis downstream of α-synuclein pathology it may be expected to prevent neuron death even in the absence of an effect on α-synuclein pathology. Therefore, the number of tyrosine hydroxylase (TH) positive neurons was also quantified through the midbrain of all mice. Both control and MLi-2 treated mice showed TH neuron loss ipsilateral to the site of injection, consistent with previous studies [23]. There was no apparent abrogation of neuron loss with LRRK2 inhibition.

**Fig. 6:**
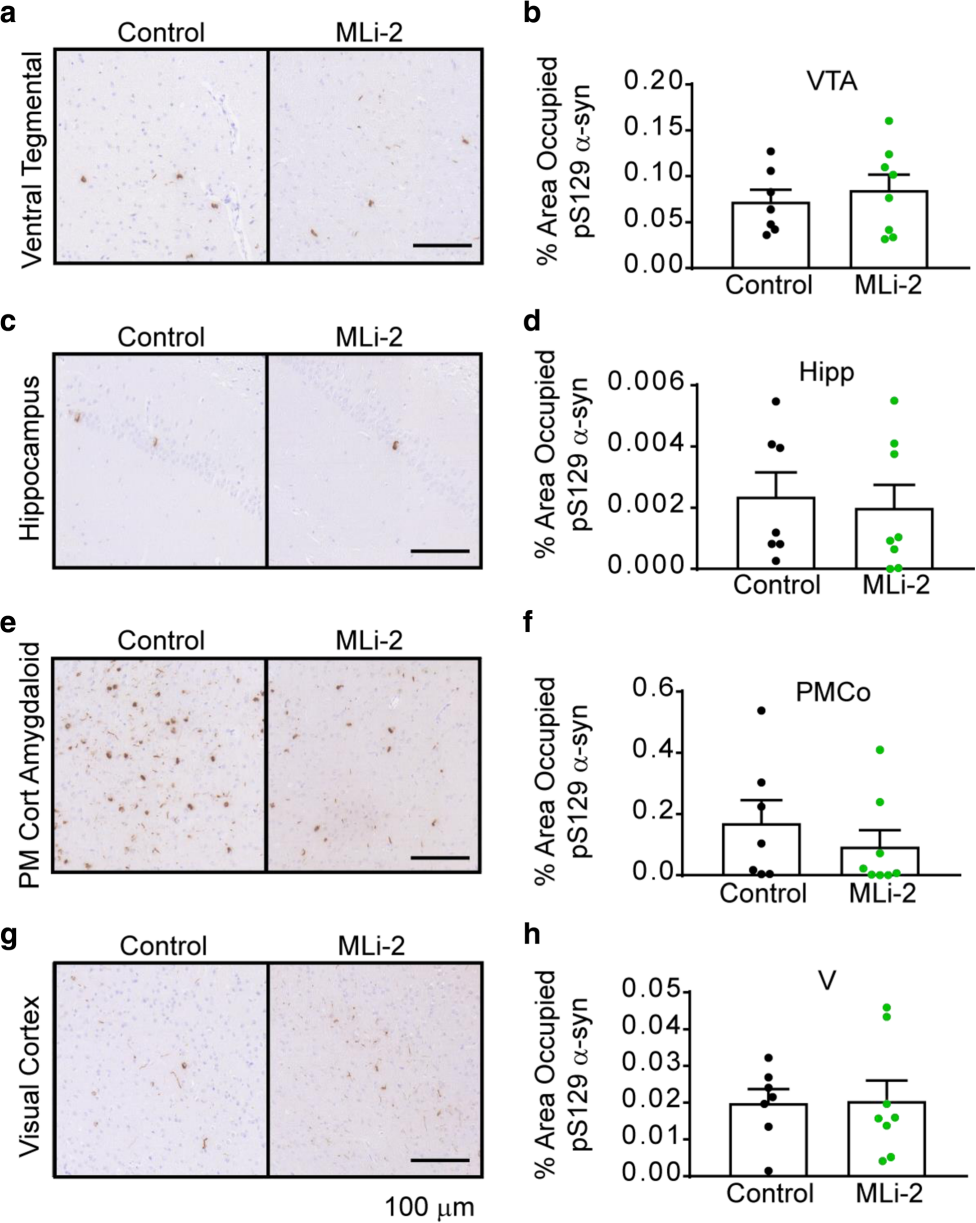
α-synuclein pathology in other regions is not altered with MLi-2 treatment. α-Synuclein pathology from 4 other regions was assayed via quantification of pS129 staining in two representative sections for each mouse. Representative staining is shown for the ventral tegmental area (**a**), the hippocampus (**c**), the posteromedial cortical amygdaloid nucleus (**e**) and the visual cortex (**g**). The quantification of the percentage area occupied with pathology is quantified for each region (**b**, **d**, **f**, **h**). All plots are means and error bars represent s.e.m. with individual values plotted. VTA *p* = 0.5671, Hipp *p* = 0.7387, PMCo *p* = 0.4142, V *p* = 0.9387, unpaired t tests

While the substantia nigra is one of the sites with the most abundant α-synuclein pathology, α-synuclein pathology spreads to many other regions, and we have previously found that G2019S mutant LRRK2 exacerbates α-synuclein pathology in a subset of these regions. To assay whether the reduction in LRRK2 kinase activity had a more concerted effect in some of these regions, we quantified pathology in four additional regions of interest: the ventral tegmental area (VTA, Fig. 6a, b), the hippocampus (Hipp, Fig. 6c, d), the posteromedial cortical amygdaloid nucleus (PMCo, Fig. 6e, f) and the visual cortex (V Fig. 6g, h). There were no significant alterations in pathology in any of these regions, although the mean pathology was reduced in the PMCo. Overall, these results suggest that the spread of α-synuclein pathology to the assayed regions is not altered.

## Discussion

Human neuropathology studies suggest that α-synuclein pathology spreads through the brain and that the spread of α-synuclein pathology to higher cortical regions correlates with the progression of cognitive decline seen in many PD patients [4–6, 19]. However, the neurobiology underlying the cell-to-cell spread of α-synuclein pathology is unknown. The predisposition of patients with mutations in *LRRK2* to get PD with commensurate α-synuclein pathology suggests that LRRK2 may regulate some aspect of PD pathogenesis. Recent cell biology studies have shown that LRRK2 may phosphorylate a subset of Rab proteins [34] and thereby regulate vesicular trafficking within the cell [10, 14, 22, 25, 26]. A role in vesicle trafficking would place LRRK2 in an ideal position to regulate cell-to-cell transmission of pathogenic α-synuclein. Further, recent studies have suggested that not only is mutated LRRK2 overactive, LRRK2 in idiopathic PD patients may also be overactive [8, 12]. Together, the putative role of LRRK2 in cellular trafficking events and the hyperactivity of LRRK2 in PD make LRRK2 an ideal candidate for kinase inhibitor development.

Work over the last 10 years has yielded several highly potent, selective and brain-penetrant LRRK2 inhibitors. One of the most potent and selective of these, MLi-2, is also bioavailable in mice and has allowed us to directly assay whether LRRK2 inhibition in vivo can modulate the formation or toxicity of α-synuclein inclusions. In this study we were able to demonstrate that MLi-2 is safe and well-tolerated by mice over a 3-month treatment period. This molecule is also able to dramatically reduce LRRK2 kinase activity. Despite this potency, LRRK2 inhibition showed no effect on the development of α-synuclein pathology in the substantia nigra, or other unrelated regions. Further, LRRK2 inhibition was unable to rescue the progressive death of dopaminergic neurons in the substantia nigra.

This is, to our knowledge, the first study to investigate whether LRRK2 inhibition is able to reverse pathogenic α-synuclein-initiated pathology and neuron death in wildtype animals. Two other reports have suggested that LRRK2 inhibition or knockdown may be efficacious in preventing PD phenotypes. In the first study, α-synuclein PFFs were used to induce α-synuclein pathology in the substantia nigra of mice and LRRK2 ASOs were administered directly into the brain ventricles of mice [39]. LRRK2 ASOs substantially reduced the number of pS129 α-synuclein inclusions and induced mild rescue of TH positive neurons in the substantia nigra. ASOs targeting LRRK2 reduced total protein levels, so it is possible that domains other than the kinase domain of LRRK2 are playing a role in α-synuclein pathogenesis that is not recapitulated by kinase inhibition. In the second study, acute administration of rotenone in rats induced both LRRK2 activation and pS129 α-synuclein accumulation in the substantia nigra [8]. LRRK2 inhibitor PF-360 was able to partially reverse this α-synuclein accumulation, but this model does not exhibit degeneration, so neuron loss was not assayed. It is also not clear if the model of α-synucleinopathy used in our study induces LRRK2 hyperactivation as was observed with rotenone treatment. It will be interesting to see in future studies whether LRRK2 hyperactivation is directly associated with α-synuclein pathogenesis or whether it is generally triggered by degeneration or another concomitant cellular pathway. In neither of these studies was there a robust rescue of dopaminergic neurons, suggesting that there is a LRRK2-independent α-synuclein pathogenesis pathway that eventually leads to neuron loss even when total pathology is reduced.

## Conclusions

There is increasing evidence that LRRK2 hyperactivation is present in both familial and sporadic PD, suggesting that LRRK2 plays a role in PD pathogenesis. If so, LRRK2 inhibitors may be therapeutic in idiopathic PD. In a mouse model of idiopathic PD, we found that LRRK2 kinase inhibitor MLi-2 was well-tolerated and showed robust target engagement. However, LRRK2 kinase inhibition in this model did not prevent motor dysfunction, α-synuclein pathology or neuron death. Future work will be important to understand whether LRRK2 inhibition is likely to be therapeutic in idiopathic PD.
